# Cell delivery systems: Toward the next generation of cell therapies for type 1 diabetes

**DOI:** 10.1111/jcmm.17499

**Published:** 2022-08-16

**Authors:** Hoang Phuc Dang, Hui Chen, Tim R. Dargaville, Bernard E. Tuch

**Affiliations:** ^1^ School of Life Science, Faculty of Science University of Technology Sydney Sydney New South Wales Australia; ^2^ School of Chemistry and Physics, and Centre for Materials Science Queensland University of Technology Brisbane Queensland Australia; ^3^ Department of Diabetes, Central Clinical School, Faculty of Medicine, Nursing & Health Sciences Monash University Melbourne Victoria Australia; ^4^ Australian Foundation for Diabetes Research Sydney New South Wales Australia

**Keywords:** cell delivery system, cell therapy, immunoprotection, type 1 diabetes, vascularization

## Abstract

Immunoprotection and oxygen supply are vital in implementing a cell therapy for type 1 diabetes (T1D). Without these features, the transplanted islet cell clusters will be rejected by the host immune system, and necrosis will occur due to hypoxia. The use of anti‐rejection drugs can help protect the transplanted cells from the immune system; yet, they also may have severe side effects. Cell delivery systems (CDS) have been developed for islet transplantation to avoid using immunosuppressants. CDS provide physical barriers to reduce the immune response and chemical coatings to reduce host fibrotic reaction. In some CDS, there is architecture to support vascularization, which enhances oxygen exchange. In this review, we discuss the current clinical and preclinical studies using CDS without immunosuppression as a cell therapy for T1D. We find that though CDS have been demonstrated for their ability to support immunoisolation of the grafted cells, their functionality has not been fully optimized. Current advanced methods in clinical trials demonstrate the systems are partly functional, physically complicated to implement or inefficient. However, modifications are being made to overcome these issues.

## 1 INTRODUCTION

Cell therapies for type 1 diabetes (T1D) traditionally involve the infusion of pancreatic islets into the portal vein of the liver with recipients taking immunosuppressive drugs to prevent the rejection of the donor tissue transplant.^1^ There are two limiting factors in this approach. They are the finite supply of pancreatic islets and the side effects of the drugs used.^2^ Pluripotent stem cells, whether embryonic or induced pluripotent, provide a potential solution to the first of these issues, with the protocol for inducing the differentiation of these cells over a period of 4 or more weeks being relatively well‐established.^3^, ^4^ Whilst producing these cells is labour intensive, strategies are being developed to mass produce them using closed laboratory systems.

The second factor limiting the clinical application of a much‐needed therapy is the toxic effects of the anti‐rejection drugs, which include an increased risk of neoplasia and infection. This limits their application in T1D cell therapies.^5^ The majority of people with T1D already get significant improvement in glycaemic control from using insulin pumps and glucose monitoring.^6^, ^7^ Only in a small number of people with labile glycaemic control and hypoglycaemic unawareness despite the use of an insulin pump and continuous glucose monitoring does the benefit of the therapy outweigh the risks. Between 1999 and 2015, there were 1086 islet transplant recipients worldwide, which is an average of just 68 per year. Of these, 30% who received islets alone became insulin independent and 20% of those who received both islets and a kidney transplant became insulin independent.^8^

**FIGURE 1:**
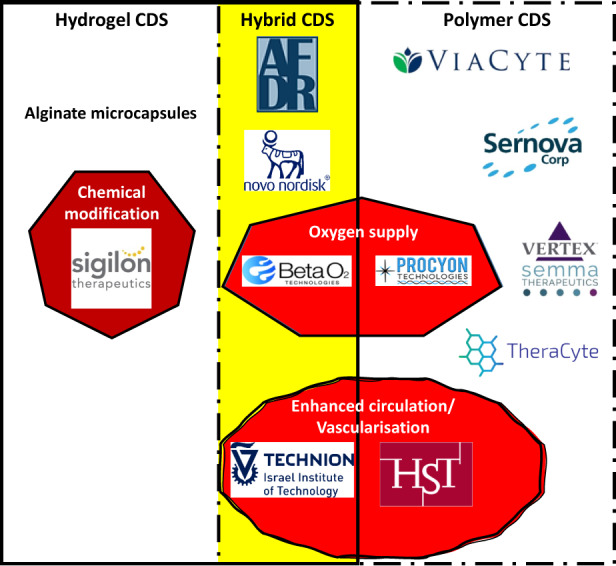
Current technology in CDS for treating T1D and representative research entities. The specifics are explained later in the review. AFDR is the Australian Foundation for Diabetes Research; HST is Health Sciences and Technology at Harvard

Strategies to deliver a cell therapy using a cell delivery system (CDS) without immunosuppression have been entertained for more than 40 years, with the first being placement of the cells inside microcapsules.^9^ The production of these devices has improved over the years, and they are usually implanted intraperitoneally in humans. Normalization of blood glucose levels has yet to be achieved, although cell function for many months has been demonstrated. Placement of these capsules in a scaffold allows them to be implanted subcutaneously—a less immunogenic site than the peritoneal cavity.^10^ Macrodevices have been produced which allow for both vascularization and immunoprotection of the enclosed ß cells, and these are usually implanted subcutaneously. Clinical trials have been conducted with these devices, but to date normalization of blood glucose levels has not been achieved. This review describes different CDS which are currently being used and documents the outcomes achieved (Figure 1). The reader will come away with a better understanding of state of the art in CDS for the treatment of T1D.

## 2 CELL DELIVERY SYSTEM IN CLINICAL TRIAL STUDIES

### 2.1 Alginate capsules as CDS

Alginate microencapsulation was the first CDS used to protect transplanted ß cells. In 1994, Shiong et al. showed that by using alginate microcapsules to transplant 15,000 pancreatic islet equivalent/kg in the peritoneal cavity of those who had previously received a kidney transplant and were therefore taking anti‐rejection drugs. The human islets produced insulin and recipients became independent of exogenous insulin for up to 9 months.^11^ However, in subsequent clinical trials with alginate microcapsules and pancreatic islets without immunosuppression, the same results could not be achieved.

In 2006, Calafiore et al. implanted intraperitoneally pancreatic islets inside alginate microcapsules in nonimmunosuppressed people with T1D.^9^ The capsules were designed with multiple coating layers to protect the cell from a host immune response. The human islets were initially placed in calcium‐alginate capsules of diameter 500‐μm and then triple coated with 0.12% and 0.06% poly‐L‐ornithine followed by 0.04% sodium alginate. Two patients received 400,000 and 600,000 islets, respectively.^9^ Insulin production confirmed by C‐peptide secretion was demonstrated for up to 1 year with a reduction in exogenous insulin requirements from 30 U to 20 U/day, and a reduction in the level of glycated haemoglobin from 10% to 7.7%. While triple coating may partially protect the clusters, it is unlikely to prevent the entry of cytokines and chemokines, which have a low molecular weight of 6–70 kDa.

In 2009, Tuch et al. also used alginate microcapsules for transplantation of human islets into the peritoneal cavity but with some modifications and achieved similar promising results.^12^ Instead of using triple coated 500‐μm calcium‐alginate microcapsules, they used single coated barium‐alginate capsules with an average diameter of 340 μm. Four patients received multiple transplants of 98,200–227,900 pancreatic islet clusters/patient. The smaller capsules and lack of coating may have improved nutrient exchange; at the same time, the encapsulated cells which survived seemed to be protected from immune attack with small amounts of insulin production, confirmed by measurement of C‐peptide, for up to 2.5 years. However, the amount of insulin released was too small to alter the blood glucose levels of the recipients. Biopsies of the grafts 16 months after implantation showed pericapsular fibrotic overgrowth.

Several companies and institutes have developed new delivery systems utilizing hydrogel, polymer and other technologies to improve the safety, viability and functionality of the implant (Table 1).

**TABLE 1:** Representative cell delivery systems in the clinical trials and preclinical studies

Clinical trial studies				
Company name	Device	Clinical trial ID	Starting date	Status
ViaCyte	Encaptra® ‐ VC‐01™	NCT02239354	12/09/2014	Terminated
		NCT04678557	22/12/2020	Active, not recruiting
	Encaptra® ‐ VC‐02™	NCT03162926	22/05/2017	Completed
		NCT03163511	23/05/2017	Recruiting
Semma Therapeutics	Semi‐permeable device	NCT04786262	08/03/2021	Recruiting
Beta O_2_	βAir	NCT02064309	17/02/2014	Active, not recruiting
Sernova	Cell Pouch™	NCT01652911	30/07/2012	Terminated
		NCT03513939	02/05/2018	Recruiting
Sigilon Therapeutics	The Shielded Living Therapeutics™	NCT04541628	09/09/2020	Suspended (Temporary enrolment halt)

Preclinical studies		
Company name	Device	Preclinical Status
TheraCyte	TheraCyte™	Have been tested in rodents and non‐human primates
Harvard‐MIT HST	ceMED	Have been tested in immunocompetent rats
AFDR	Pre‐vascularized melt electrowritten device	Have been tested in immunocompetent mice
Novo Nordisk	Electrospun nanofibrous device	Have been tested in immunocompetent mice
Procyon Technologies	miniaturized electrochemical oxygen generator combined with TheraCyte™	In discovery phase
Technion	Bioengineered vascular bed	Have been tested in immunodeficient mice

### 2.2 Encaptra® device from ViaCyte

Rather than using microcapsules the company ViaCyte Inc developed a semipermeable pouch called Encaptra®which was implanted subcutaneously. It contained human embryonic‐stem‐cell‐derived pancreatic precursor cells (PEC‐01™), which had been shown previously to differentiate into mature ß cells and normalize blood glucose levels of recipient diabetic mice.^13^ In 2014, the company commenced its first clinical trial in people with T1D (ClinicalTrials.gov Identifier [CI]: NCT02239354), using this cellular device termed VC‐01™ and without giving recipients anti‐rejection drugs.^14^ The device was found to be safe, but there was a host reaction against the implant resulting in necrosis of most of the cells due to hypoxia. Small areas of the graft were vascularized, but too few for evidence of cellular function to be elicited.^15^

**FIGURE 2:**
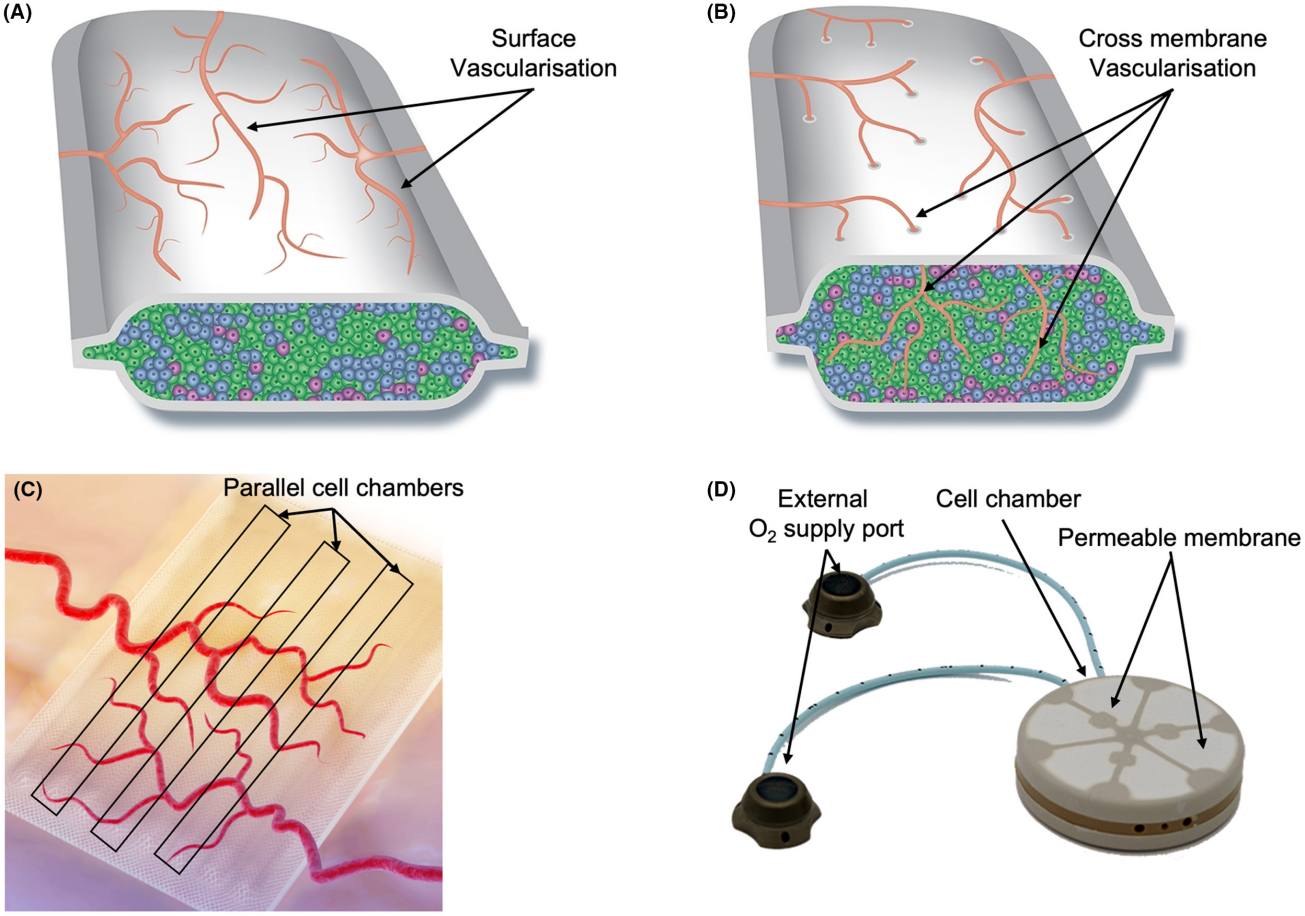
Representative CDS used in clinical trials. A: ViaCyte PEC‐Encap; B: ViaCyte PEC‐Direct; C: Sernova Cell Pouch™; D: Beta O_2_ βAir. Pictures used with the permission of ViaCyte, Beta O2 and Sernova

In 2017, ViaCyte modified the device with multiple large across‐membrane pores to support vascularization but dispensing with an immuno‐isolatory effect; the seeded device was called VC‐02™ or PEC‐Direct (Figure 2B). In its second and third clinical trials (CI: NCT03162926, NCT03163511)^16^ with this device, ViaCyte gave recipients Tacrolimus and Mycophenolate Mofetil to prevent rejection of the implanted cells. Because of the risk of toxic side effects from these drugs, T1D recipients were restricted to those who had hypoglycaemic unawareness. These trials were more successful than the initial one, with the majority of the 15 recipients developing an increase in fasting C‐peptide levels and development of a mixed meal stimulated C‐peptide secretion by 6–9 months after the PEC‐01 cells were implanted. Exogenous insulin requirements were reduced by 20% during the 1 year follow‐up, and glycaemic control was maintained with the level of glycated haemoglobin <7%.^17^, ^18^ Presumably, an increase in the number of cells implanted is needed to achieve exogenous insulin independence.

To try and eliminate the need for the use of immunosuppressants, ViaCyte subsequently modified its PEC‐Encap device, in conjunction with the material science company W.L. Gore and Associates.^19^ The new device, made from expanded polytetrafluoroethylene (ePTFE), has both immuno‐isolatory and pro‐angiogenic properties (Figure 2A).

With this device, ViaCyte commenced a fourth clinical trial (CI: NCT04678557) in 2020 using up to 12 PEC‐Encap devices per recipient, who did not take anti‐rejection drugs. The trial also included sentinel implants to be removed for histological analysis over a period of 26 weeks; however, the results are not yet available.^20^

Besides using the bioengineered device PEC‐Encap to minimize or prevent graft rejection by the host, ViaCyte in collaboration with CRISPR Therapeutics is introducing gene editing to prevent rejection of cells to be implanted. They are utilizing CRISPR‐Cas9 gene editing technology to create human embryonic stem cell clones that lack the β2‐microglobulin (B2M) gene and express a transgene encoding programmed death‐ligand (PD‐L1).^15^ The lack of B2M is thought to prevent the attachment of host immune cells and the expression of PD‐L1 may inhibit the activation of autoimmune T‐cells.^21^ In 2022, ViaCyte and CRISPR Therapeutics commenced this new trial.^22^

### 2.3 Semipermeable device from Semma Therapeutics

Semma Therapeutics, acquired by Vertex Pharmaceuticals in 2019, is the first company to use fully differentiated stem cell derived islet cell clusters in clinical trials. Semma Therapeutics planned to use the cells inside a device and implant it subcutaneously. The device is formed from two semipermeable polyvinylidene fluoride membranes, in which the cells are introduced between the membranes. Cross‐membrane channels are distributed throughout the device to enhance vascularization. The molecular weight cut‐off of the device is ~500 kDa,^23^ which means that immune cells and IgM antibodies cannot gain entry; however, IgG antibodies have a molecular weight of around or below 150 kDa, and cytokines should be able to permeate. Regardless, the human cells in the implant showed production of insulin for up to 80 days in a large preclinical trial in non‐diabetic pigs.^24^ Despite these encouraging results, the company changed direction in 2021 and took the conventional approach used with human pancreatic islets in its first clinical trial with fully differentiated stem cell derived ß cells VX‐880, formerly STx‐02 (CI: NCT04786262). The company first wished to determine that the ß cells could function when infused into the portal vein of people with T1D taking anti‐rejection therapy.^25^ Vertex has achieved its goal at least for the first recipient who was previously C‐peptide negative. In October 2021, it reported the production of large amounts of C‐peptide (280 pmol/L) 90 days after an infusion of ß cells, in association with a reduction of glycated haemoglobin from 8.6% to 7.2%, and a 91% reduction of exogenous insulin requirements.^25^

### 2.4 βAir device from Beta O_2_

The next CDS we wish to discuss is the βAir device (Figure 2D) from Beta O_*2*_ (Israel). The device is different from other CDS in that it houses cells in an immunoisolatory manner as well as allows for a continuous supply of exogenous oxygen for the enclosed ß cells which thrive in an oxygen rich environment.^26^ The cells are placed in a flat slab of alginate of high guluronic acid content and separated from the external environment by a semipermeable hydrophilic membrane made of PTFE, of pore size 0.4 μm, incorporated with alginate having a high mannuronic acid content.^27^ It is the membrane‐alginate complex that provides partial immunoisolation from the host. For the first clinical trial with this device, which commenced in 2014 (CI: NCT02064309), it was seeded with human pancreatic islets and implanted subcutaneously in people with T1D. Small amounts of insulin were produced by the enclosed islets for up to 8 weeks post implantation, but not at levels allowing the reduction of required exogenous insulin.^26^ It is technically possible to increase the number of pancreatic islets in the device,^28^ whether from donor pancreases or stem cells, but this has yet to be trialled in humans. There is a practical disadvantage of using a device with the continuous need for an external oxygen supply, because of an increased risk of infection, despite the function of encapsulated islets being optimized by doing so.^29^

### 2.5 Cell pouch™ device from Sernova

Another device that has been used in clinical trials for the treatment of T1D is the Cell Pouch™ (Figure 2C) made by Sernova. The initial goal of this device was to provide pre‐vascularized chambers for islet transplantation, but without immunoprotection to the islets introduced into its chambers. Cell Pouch™ is a rectangular microporous pouch made of polypropylene membranes. Instead of having a single cell chamber like the other devices described above, the body of the Cell Pouch™ is divided into multiple parallel cylindrical chambers which are prefilled with solid plugs made of PTFE. After implantation and prevascularization have been achieved, the PTFE plugs are removed, leaving empty space for the introduction of islets.^30^ In the first clinical study with these devices, conducted in 2012 (CI: NCT01652911), they were implanted subcutaneously in three people with T1D for a median of 53 days. The PTFE plugs were then removed, pancreatic islets at a dose of ~6000 IEQ/kg introduced, and immunosuppression of recipients initiated. Histological study of the device at 6 weeks after being inserted subcutaneously showed neovascularization. Within 24 h of the islets being implanted, there was a spike of C‐peptide release, but none thereafter, indicating the subsequent failure of the graft.^30^

In the company's second clinical study with the device, which commenced in 2018 (CI: NCT03513939), immunosuppression of recipients was initiated a month after implantation, with the first introduction of islets a further month later. In July 2019, Sernova reported promising results from the first recipient with no major adverse events and the device being well vascularized.^31^ Stimulated C‐peptide has been reported for up to 9 months in 2 recipients with a lowering of the level of glycated haemoglobin from 10.6% to 7.6% and a reduction in daily exogenous insulin from 49 U to 28 U.^32^ Recognizing that it would be helpful if recipients did not have to be immunosuppressed, Sernova is now contemplating placing the ß cells in hydrogel capsules to protect them from the host immune system once they are introduced into the pre‐vascularized device.

### 2.6 The Shielded Living Therapeutics™ from Sigilon Therapeutics

**FIGURE 3:**
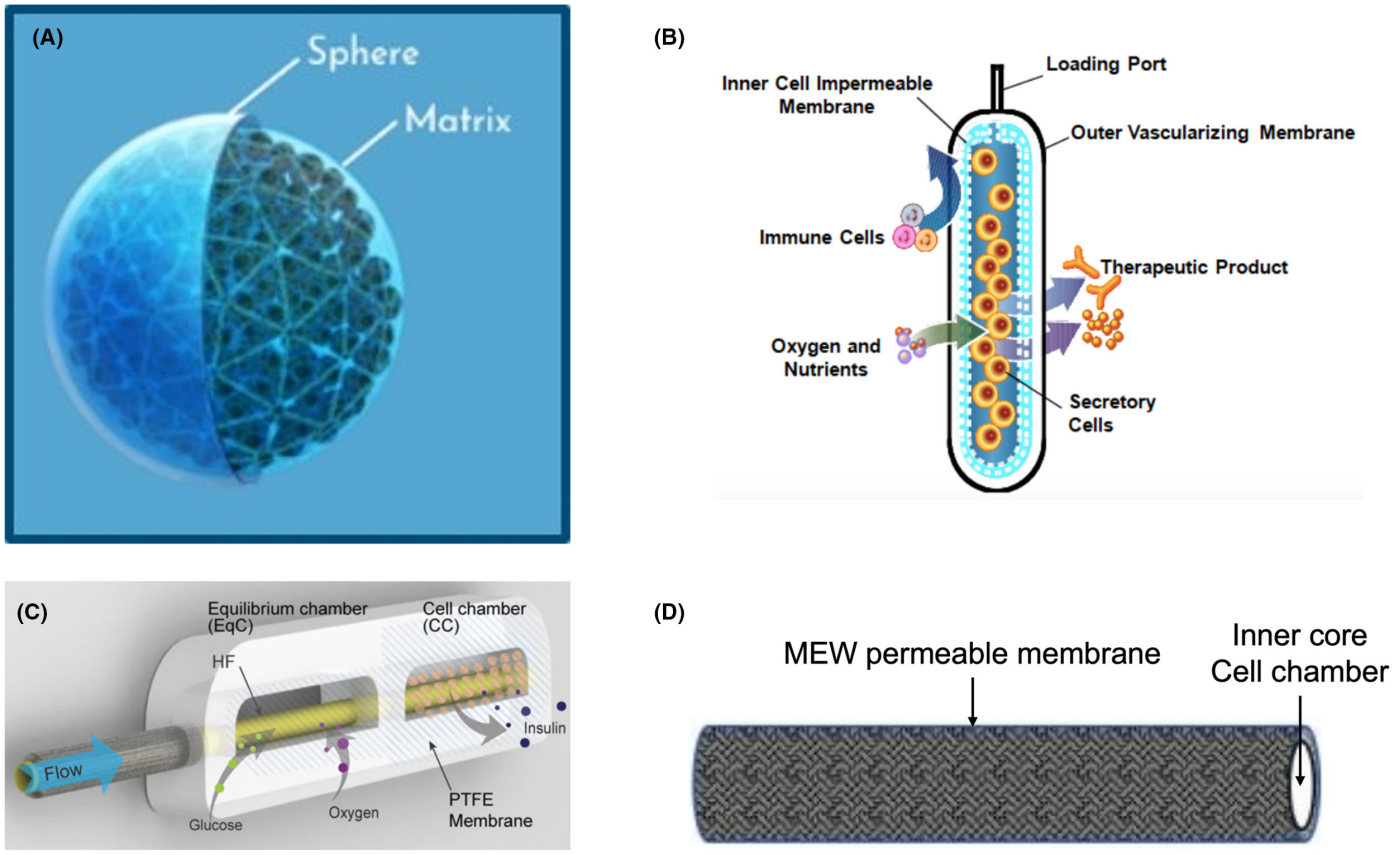
Representative CDS used in pre‐clinical studies. A: Sigilon Shielded Living Therapeutics™; B: TheraCyte™; C: HST convection enhanced device; D: AFDR pre‐vascularized MEW scaffold.^10^ Pictures used with the permission of Sigilon, TheraCyte and HST

Another promising technology under clinical study is the Shielded Living Therapeutics™ sphere (SLTx, Figure 3A) of Sigilon Therapeutics Inc., now allied with Eli Lilly. The spheres of diameter 1.5 mm consist of an inner core of cell clusters in a modified alginate matrix and an external coating of alginate modified with the chemical triazole‐thiomorpholine dioxide (TMTD)^33−^. The modification makes the capsules resistant to most of the host fibrotic reaction that adversely affects devices.^33^ In its preclinical study with immunocompetent diabetic C57BL6 mice, encapsulated human stem cell‐derived ß cells introduced into the peritoneal cavity immediately normalized blood glucose levels and maintained normoglycaemia until their removal 174 days later.^34^ In taking this device to the clinic, Sigilon initially chose the non‐autoimmune model of haemophilia, a blood clotting disorder, rather than autoimmune TID to test the safety and efficacy. The clinical study was commenced in 2020 (NCT04541628) using SIG‐001 spheres containing allogeneic cells genetically manipulated to express human factor VIII.^35^, ^36^ However, the trial was placed on hold in July 2021 because of safety concerns, with the third recipient, who received the highest number of cells, developing antibodies to Factor VIII.^37^ The company is investigating if there is a connection between TMTD and the development of the inhibitors. Whether the TMTD microcapsules will be used to introduce ß cells into TID recipients will require the above safety concerns to be overcome.

## 3 CELL DELIVERY SYSTEMS IN PRECLINICAL STUDIES

Below we have listed several different preclinical strategies being used to develop CDS as a therapy for T1D.

### 3.1 TheraCyte™ device from TheraCyte

TheraCyte™ (Figure 3B) is a cell microencapsulation system from TheraCyte Inc. As with most other CDS, this device is designed to both protect the transplanted cells from immune attack and enhance neovascularization. The device is flat rectangular in shape, 4 cm in length, 1 cm in width and covered by 3 layers.^38^ The outer layer is made of woven polyester mesh to provide physical protection. The middle and inner layers are made of PTFE membranes manufactured by W.L. Gore and Associates. The middle layer has pore sizes of 5 μm and supports neovascularization, while the inner layer has a much smaller pore size of 0.4 μm to provide immune protection. The device has been seeded with human β‐cells and implanted subcutaneously in diabetic immunodeficient rodents,^39^ and with the monkey and porcine islets in partially pancreatectomized non‐human primates, but not in diabetic humans.^40^, ^41^ Normalization of blood glucose levels was achieved in the diabetic mice,^39^ and ß cell survival and function for up to 12 months in the rhesus monkeys.^41^ A major concern with the device is the host fibrotic reaction to it, thereby adversely affecting the function of the implanted cells. This was observed in a clinical study in 2000 involving subcutaneous auto‐ and allo‐transplantation of parathyroid tissue for the treatment of hypoparathyroidism in the absence of immunosuppression.^42^ Between 8 and 14 months after the device with parathyroid tissue was implanted, the implants were surrounded by fibrotic tissue with no increase in the level of parathormone.^42^

### 3.2 Convection enhanced device from Harvard‐MIT Health Sciences and Technology

In 2021, Yang et al. introduced a convection‐enhanced microencapsulation device (ceMED, Figure 3C) to support cell viability and provide immunoprotection.^43^ The device made of PTFE consists of two equal sized chambers of 1–2 cm in length, an equilibrium chamber to improve nutrient exchange and a cell chamber, through the centre of both of which is a hollow fibre for pump induced flow of culture medium. The pore size of the external membrane of the device is 10 μm and that of the membrane surrounding the cell chamber 0.2 μm for immune protection. The molecular weight cut‐off of the membrane surrounding the hollow fibre is 100 kDa. By using ceMED to implant MIN6 cells in diabetic immunocompetent rats, the investigators were able to reduce the blood glucose levels of diabetic mice from 600 mg/dL to 200 mg/dL in 5 days and maintain this level for up to 30 days. There are no reports we could find of this device being used with human ß cells. Major scale up would be needed for this and other CDS to house the large number of human ß cells needed for clinical use.

### 3.3 Pre‐vascularized melt electrowritten scaffolds from the Australian foundation for diabetes research

Mridha et al. has developed a device which combines alginate microencapsulation, which provides some immunoprotection, with pre‐vascularization of 3D melt electrowritten (MEW) scaffolds made of polycaprolactone (Figure 3D).^10^ In the 2020 paper, cylindrical scaffolds of diameter 2 mm and length 2 cm and pore size varying from 50 to 200 um were implanted subcutaneously in diabetic immunocompetent mice for 2 weeks to allow vascularization. Thereafter, encapsulated mouse islets were introduced into the device, with normalization of blood glucose levels achieved in allografted mice within 48 h and maintained for up to 105 days. This is in contrast to the lack of glucose normalization when encapsulated mouse islets placed in MEW scaffolds were implanted without pre‐vascularization. Immunohistochemical analysis showed minimal fibrosis surrounding the capsules, thereby allowing the islets to function. The next step for this CDS is to optimize the design and number of cells needed for translation into clinical trials.

### 3.4 Electrospun nanofibrous encapsulation device from Novo Nordisk

**FIGURE 4:**
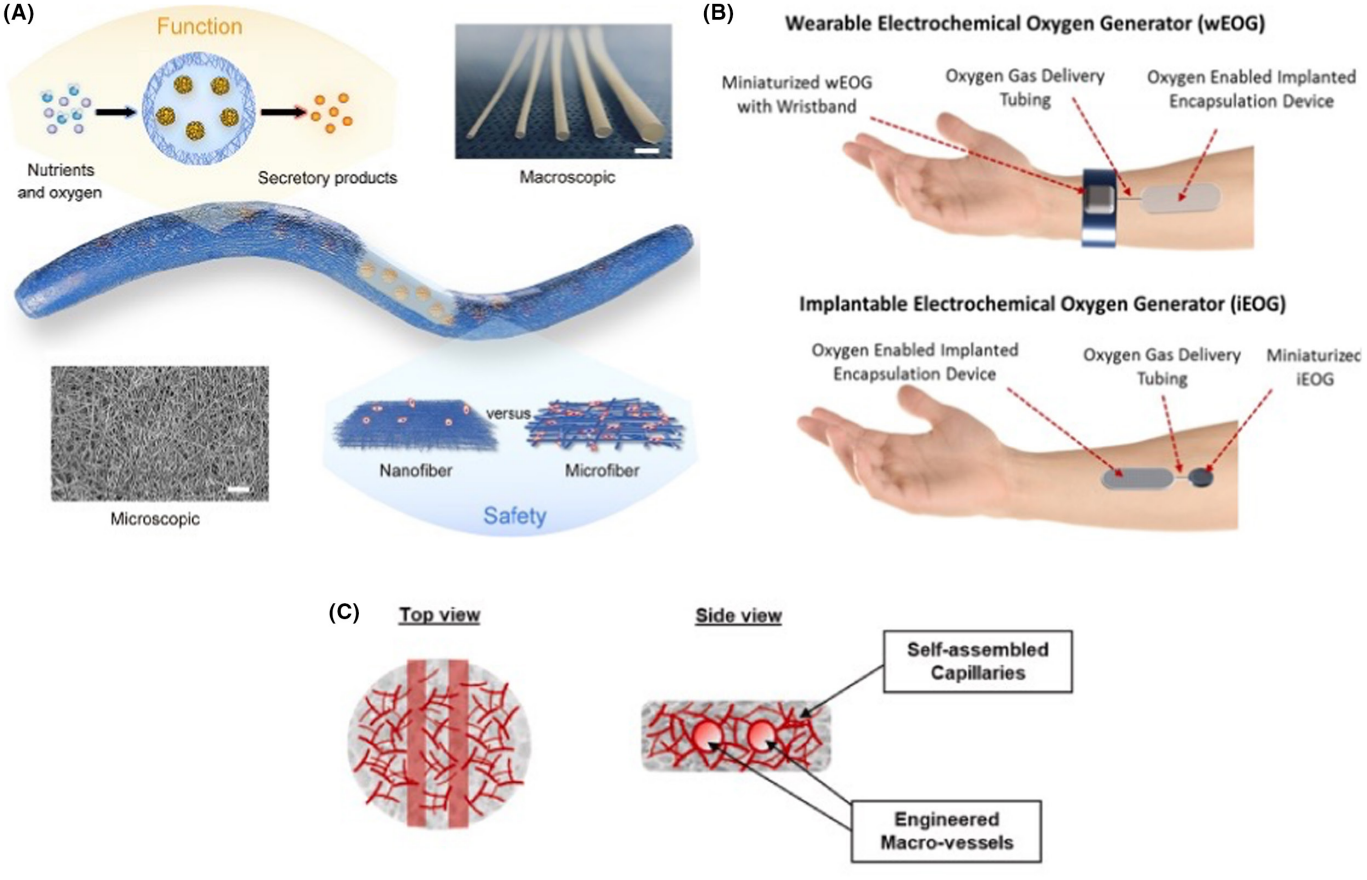
Representative CDS used in pre‐clinical studies. A: Novo Nordisk Electrospun nanofibrous encapsulation device; B: Procyon oxygenation cell delivery device;^45^ C: Technion vascular bed platform.^47^ Pictures used with the permission of Novo Nordisk

In 2021, a project at Cornell University, funded by Novo Nordisk, also utilized both 3D printed scaffolds and alginate hydrogels such as Mridha et al., yet with different customization and without pre‐vascularization.^44^ Wang et al. fabricated electrospun tubular scaffolds from medical‐grade thermoplastic silicone‐polycarbonate‐urethane (TSPU) with a diameter of 0.5–3 mm, fibre size of ~0.27 μm and pore size of ~1 μm (Figure 4A). Human stem cell‐derived β‐cell clusters were dispersed in alginate solution, injected into the centre of the cylindal scaffolds (1250 clusters/scaffold) and crosslinked in CaCl_2_ solution. The devices were implanted subcutaneously in both immunodeficient and immunocompetent diabetic mice with normalization of blood glucose levels within the first few weeks. While the blood glucose levels remain normal for up to 120 days in immunodeficient mice, the same was achieved for only up to 60 days in immunocompetent mice. The research group is attempting to scale up the device for use in dogs.

### 3.5 Oxygenation cell delivery device from Procyon Technologies

In 2019, following the strategy used by Beta Oxygen of providing an external oxygen supply to improve cell viability and graft function, Procyon Technologies developed a miniaturized electrochemical oxygen generator to be worn as a wristband (Figure 4B).^45^ The generator will generate and provide oxygen to the cell clusters via oxygen delivery tubing that connects the generator with the cell chamber of the implanted device. The company's researchers wish to use it as a permanent oxygen source for transplanted ß cell clusters placed in a Theracyte™ device. One of the drawbacks of this device is that the implant needs to be in close proximity to the wristband, hence limiting the implant volume and also may affect hand movement. Because of that, the company's next target is to reduce the size of the oxygen generator to an implantable size so that it can be implanted with the Theracyte™ device, which gives more options for choosing the site of implantation. This strategy could assist in improving cell viability and avoiding hypoxic necrosis in CDS.

### 3.6 Multi‐cell vascular bed for tissue implantation from Technion

A novel approach being pursued by the group of Levenberg is the bioengineering of a vascular bed, to enhance the integration between the implant construct and the native vasculature, with the aim of improving cell survival and cell functioning.^46^ The bed has polymer and cell components. The polymers consist of polylactic‐co‐glycolic acid and polylactic acid in a 1:1 ratio with a resultant pore size of 300–600 μm and porosity of 93%. The cells seeded onto the scaffold sponge are a mixture of human vascular endothelial cells (HUVEC) and human fetal fibroblasts in a ratio of 50,000: 30,000 HFFs: 1 islet in 15 μl seeding medium. In this elegant study, HUVECs rearrange themselves and provide connections among all cells in the bed within 10 days. Implantation of a scaffold with 50 mouse islets in a diabetic nude mouse showed a connection with the native vasculature after 14 days. Blood glucose levels were normalized after 18 days and maintained for at least a further 28 days, with cell viability of 80%. To improve the results, the same group reports the use of a moulding technique to create macro scale vessels with an inner diameter of 600 μm that run through the platform resulting in dual scale vessel supporting constructs (Figure 4C).^47^ It is reasonable to assume human islets will behave similarly to mouse islets, although there are no reports of such experiments. Moreover, the multi‐cell vascular bed will not result in immuno‐isolation of the islets if allografted.

## 4 CHARACTERISTICS OF DIFFERENT CELL DELIVERY SYSTEM TECHNOLOGY

Comparing the characteristics of different techniques used for cell therapy in T1D (Table 2), one finds that the hydrogel systems share common benefits. They are easy to prepare (do not require advanced tools or in vitro culture preparation) and easy to implant. Among hydrogel capsules system, the simplest form (340 μm alginate capsules) provides the best nutrient exchange for the islets and maintains viability for up to 2.5 years.^12^ However, glucose response was minimal and there was no therapeutic effect.^12^ Furthermore, a limit of using hydrogel capsules is the inability to retrieve them if needed, which poses a risk to the patients if there are any complications related to the implant.

**TABLE 2:** Characteristics of different cell delivery system technologies and their therapeutic effects in clinical trials

Main system	Supporting technology	Immune protection mechanism	Cell support mechanism	Complexity	Therapeutic effectiveness in clinical trial studies
Hydrogel	None	Physical barrier from hydrogel	None	Easy to prepare; Easy to implant; Cannot retrieve	Islets survived up to 2.5 years; no therapeutic effect^12^
	Anti‐rejection drug	Physical barrier from hydrogel and anti‐rejection drug			Exogenous insulin independence for up to 9 months^11^
	Coating	Physical barrier from multiple coating and hydrogel			Reduction of exogenous insulin dosage from 30 U/day to 20 U/day for up to 1 year^9^
	Chemical modification	Chemical barrier			Unknown. Clinical trial has been put on hold^33^, ^34^
Polymer	None	Physical barrier from semi‐permeable membrane	Semipermeable membrane supports vascularization	Require a specialized tool to prepare the device; Easy to implant and retrieve	Semma's device can reduce 91% exogenous insulin requirement in the first 90 days^25^; Sernova's device can reduce daily exogenous insulin requirements from 49 U to 28 U for up to 9 months^32^
	Oxygen supply		Wearable permanent oxygen supply device	Require a specialized tool to make device; Limited hand movement and cell loading capacity; Hard to implant and retrieve	In Discovery phase^45^
	Enhanced circulation/vascularization		Fluid circulation system	Require specialized tool to prepare the device; Easy to implant and retrieve	In Preclinical phase^43^
Hybrid	None	Physical barrier from semi‐permeable fibrous membrane/polymer matrix and from hydrogel	Semipermeable membrane supports vascularization	Require a specialized tool to prepare the device; Easy to implant and retrieve	In Preclinical phase^10^, ^44^
	Oxygen supply		Oxygen chamber	Require a specialized tool to make the device; Complex to implant and retrieve; Require frequent external supply of oxygen	Islets survive for up to 8 weeks; No therapeutic effects ^26^
	Enhanced circulation/vascularization		Pre‐vasculature network	Require in vitro preparation prior to implantation; Easy to implant and retrieve	In Preclinical phase^46^, ^47^

The use of multiple hydrogel coating seems to maintain the function of transplanted islets with some efficacy (reduction of exogenous insulin requirement) by offering better protection from host immune system.^9^ However, multiple coatings increase the thickness and limit nutrient exchange, which results in the shortening of cell viability from 2.5 to 1 year.^9^ Chemically modified alginate allows for a smaller diameter capsule with improved nutrient exchange, while providing a chemical barrier to immunoprotect the islets.^33^, ^34^ However, there are no clinical results as yet.

Compared with the hydrogel systems, the polymer systems have the advantage that they are able to support cell viability via enhanced vascularization or oxygen supply. Another benefit of using a polymer system is that most of the devices are easy to implant and retrieve when needed because the islets are contained within them. Among the companies using polymer systems for cell delivery, Semma Therapeutics and Sernova have demonstrated the therapeutic effect of the implants in humans. Semma's device without pre‐vascularization can reduce the required exogenous insulin in a human for up to 90 days.^48^ Sernova's device, with pre‐vascularization, can reduce the required exogenous insulin in human for up to 9 months.^49^ Further improvement using a permanent oxygenation source^45^ and enhanced fluid circulation system^50^ may improve cell viability, nutrient exchange and glucose responsiveness, but research is still in the preclinical stage.^51^

In between the hydrogel and polymer systems are hybrid systems, which utilize both hydrogels and polymer systems for protection and support. Instead of using polymer membranes for immune protection, the hybrid system uses them to prevent fibrotic reactions surrounding the alginate capsules and support vascularization, while the hydrogel is used for immune protection.^52^, ^53^ Among the hybrid system, only βAir device of Beta O_2_ has been used in a clinical trial.^26^ With the supply of oxygen from an oxygen chamber, the implanted cells can survive for up to 8 weeks, yet no therapeutic effect was observed.^26^ The other systems using the hybrid system only or combined with multiple‐cell culture to create a pre‐vasculature bed are still in preclinical stages.^53^, ^54^, ^56^

## 5 SUMMARY

Encapsulation of cells in alginate has been used as a CDS with or without anti‐rejection drugs for several decades. Limiting the success of the microcapsules is the host fibrotic reaction to them, thereby limiting the passage of nutrients and waste products. To improve the protection and enhance the function of the graft, multiple approaches have been developed. These include transplantation subcutaneously rather than in the peritoneal cavity, immune protection with a physical barrier, immunomodulation using modified materials or gene edited cell clusters, pre‐vascularization and hyperoxygenation.

## 6 PERSPECTIVES

There is reason to believe that the next generation of CDS for treatment of T1D will be a hybrid system containing several features but without the need for anti‐rejection medicines and the inherent risks it brings. Only by doing so will it be possible to safely treat most people with T1D using the seemingly inexhaustible supply of stem cell‐derived ß‐cell clusters.

## AUTHOR CONTRIBUTIONS

Hoang Phuc Dang involved in conception and design, data analysis and interpretation and manuscript writing. Hui Chen and Tim Dargaville written the manuscript. Bernard E Tuch involved in conception and design, manuscript writing and final approval of the manuscript.

## AUTHOR CONTRIBUTIONS

**Dang HP:** Conceptualization (equal); data curation (lead); formal analysis (lead); investigation (lead); visualization (lead); writing – original draft (lead); writing – review and editing (equal). **H Chen:** Conceptualization (equal); supervision (equal); writing – original draft (equal); writing – review and editing (equal). **Dargaville TR:** Writing – review and editing (equal). **Tuch BE:** Conceptualization (equal); funding acquisition (lead); resources (supporting); supervision (equal); writing – review and editing (equal).

## CONFLICTS OF INTEREST

The authors acknowledge that there is no conflict of interest.

## Data Availability

This review article is exempt from Data sharing
